# Molecular Dynamics of a Thermostable Multicopper Oxidase from *Thermus thermophilus* HB27: Structural Differences between the Apo and Holo Forms

**DOI:** 10.1371/journal.pone.0040700

**Published:** 2012-07-10

**Authors:** Martiniano Bello, Brenda Valderrama, Hugo Serrano-Posada, Enrique Rudiño-Piñera

**Affiliations:** Departamento de Medicina Molecular y Bioprocesos, Instituto de Biotecnología, Universidad Nacional Autónoma de México, Cuernavaca, Morelos, México; University of Akron, United States of America

## Abstract

Molecular dynamic (MD) simulations have been performed on *Tth*-MCO, a hyperthermophilic multicopper oxidase from *thermus thermophilus* HB27, in the apo as well as the holo form, with the aim of exploring the structural dynamic properties common to the two conformational states. According to structural comparison between this enzyme and other MCOs, the substrate in process to electron transfer in an outer-sphere event seems to transiently occupy a shallow and overall hydrophobic cavity near the Cu type 1 (T1Cu). The linker connecting the β-strands 21 and 24 of the second domain (loop (β21–β24)_D2_) has the same conformation in both states, forming a flexible lid at the entrance of the electron-transfer cavity. Loop (β21–β24)_D2_ has been tentatively assigned a role occluding the access to the electron-transfer site. The dynamic of the loop (β21–β24)_D2_ has been investigated by MD simulation, and results show that the structures of both species have the same secondary and tertiary structure during almost all the MD simulations. In the simulation, loop (β21–β24)_D2_ of the holo form undergoes a higher mobility than in the apo form. In fact, loop (β21–β24)_D2_ of the holo form experiences a conformational change which enables exposure to the electron-transfer site (open conformation), while in the apo form the opposite effect takes place (closed conformation). To confirm the hypothesis that the open conformation might facilitate the transient electron-donor molecule occupation of the site, the simulation was extended another 40 ns with the electron-donor molecule docked into the protein cavity. Upon electron-donor molecule stabilization, loops near the cavity reduce their mobility. These findings show that coordination between the copper and the protein might play an important role in the general mobility of the enzyme, and that the open conformation seems to be required for the electron transfer process to T1Cu.

## Introduction

Multicopper oxidases (MCOs) belong to a ubiquitous family of metalloproteins involved in lignin degradation, copper homeostasis and iron metabolism [1]–[3]. In some cases they present catalytic activity as oxidoreductases coupling the reduction of O_2_ to H_2_O, with the oxidation of different substrates [1]–[4]. MCOs can be separated into two groups by substrate specificity. The first group catalyzes the oxidation of organic substrates and transition metal ions (laccases and ascorbate oxidase) [5], [6] and the second group cannot oxidize organic substrates (Fet3p, ceruloplasmin, CueO, MnxG) [7]–[9]. In both groups catalysis occurs following an outer-sphere electron transfer mechanism, in which the substrate and the enzyme do not necessarily establish chemical bridges [10].

**Figure 1:**
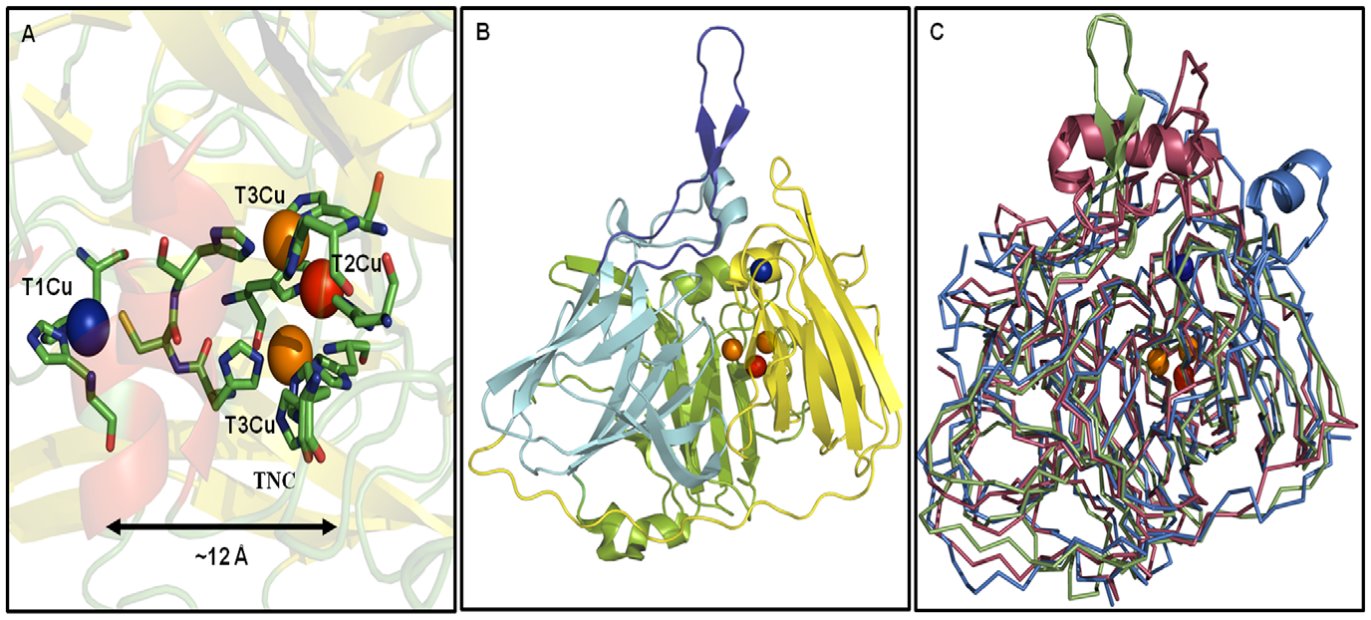
Structural topology of holo-*Tth*-MCO and superposition with others MCÓs. A) Multinuclear metal site of the holo-*Tth*-MCO. B) Structural depiction of the three cupredoxin domains of holo-*Tth*-MCO, (domain 1, green), (domain 2, light blue) and (domain 3, yellow), the loop (β21–β24)_D2_ is in deep blue cartoons. C) Ribbon superposition of the holo forms of *Tth*-MCO (green), CueO (red, PDB entry 1N68) and CotA (blue, PDB entry 1UVW), the main differences among them are represented in cartoon structure. Mononuclear copper center (T1Cu) is shown in blue and trinuclear copper cluster (TNC) is shown in orange (T3Cu) and red (T2Cu) Van der Waals representation.

MCOs contain four copper ions arranged in two centers. The first one is a mononuclear type 1 center (T1Cu) where the copper ion is coordinate by two histidine and one cysteine residues, being the latter bond the responsible for an intense adsorption band around 600 nm, giving the enzyme a blue color. The T1Cu is also distinguished by a narrow parallel hyperfine splitting in its electron paramagnetic resonance (EPR) spectrum. The second center is a trinuclear copper cluster (TNC) composed of a type 2 copper ion (T2Cu) bound to two histidine residues and characterized by a typical EPR signal, but not detectable in the UV–visible region and a type 3 (T3Cu) or binuclear copper site where each copper ion is bound to three histidine residues. The T3Cu center is characterized by a shoulder at a wavelength of 330 nm and with no EPR signal due to the presence of a strongly antiferromagnetic coupled Cu^II^ pair that is hydroxide bridged [2]. In the currently accepted reaction mechanism, the substrate is first oxidized by the T1Cu site, which then shuttles electrons over ∼ 12 Å to the TNC, where O_2_ reduction takes place (fig. 1A) [11].

MCO from *Thermus thermophilus* HB27 (*Tth*-MCO) is a 48.7 kDa thermostable enzyme with oxidase activity towards ferrous ions and organic compounds such as 2,6-dimethoxy phenol, syringaldazine and ABTS. The optimal reaction temperature of this thermostable laccase is ∼92°C, with a half-life of thermal inactivation at 80°C of over 14 h, making it the most thermostable MCO reported so far([12], Kevin Mendez-Acevedo & Brenda Valderrama, unpublished data). Its structural topology is comprised of three cupredoxin domains, with a mononuclear copper centre localized in the third domain, and a trinuclear copper center (TNC) located between the first and third domains (fig. 1B). Despite the fact that structural information about this protein is available for apo-*Tth*-MCO as well as for holo-*Tth*-MCO (2XUW and 2XU9 PDB’s, respectively), little information is known about its conformational mobility [13].

Structural comparison between apo-*Tth*-MCO and holo-*Tth*-MCO shows a root mean square deviation (RMSD) of 0.014 nm, suggesting that no significant conformational changes accompany the copper coordination. On the other hand, it has been reported that copper site geometry is somehow modulated by the overall protein scaffold [14]. In addition, unfolding studies have shown that the interplay between the metal center and the polypeptide chain determines the stability of holoproteins [15], [16]. Coupled with this, structural comparison between *Tth*-MCO and others MCOs with which it shares highest structural homology, point out that the main structural differences are situated close to the T1Cu site and the proposed electron-transfer site (fig. 1C). For *Tth*-MCO the linker connecting the β-strands 21 and 24 of the second domain (loop (β21–β24)_D2_) residues 287–312), has the same conformation in both crystal structures, and forms a lid at the entrance of the electron-transfer cavity (Fig. 1B). Loop (β21–β24)_D2_ has been tentatively assigned a role in the enzyme mechanism: it seems to occlude access to the electron-transfer site. Furthermore, either the conformation orientation of this loop or the chemical nature of the electron-transfer process could be responsible for the frustration in attempts by our research group, to crystallize the complex between holo-*Tth*-MCO and ABTS (2,2′-azino-bis(3-ethylbenzothiazoline-6-sulphonic acid).

Along this line, this enzyme is an interesting model for carrying out molecular dynamical studies, because is one of the few MCOs determined in its apo and holo forms [13]. MD simulation studies on copper proteins have shown that a higher RMSD is expected for the apo form when this is derived from the holo form [17], causing confused information about the main motions associated with each conformational state. Furthermore, more MD simulation studies are needed on MCOs in order to gain insights into the dynamic behavior of these systems. To date, most MD simulation research to study conformational mobility linked to copper coordination, has been focused mainly on cupredoxins.

In this work, classical MD simulations were carried out for apo-*Tth*-MCO and holo-*Tth*-MCO, in order to shed more light onto the conformational dynamics of loop (β21–β24)_D2_, as well as to compare basic features of the structure and dynamics and how copper coordination affects the protein matrix. In general, it is found that the two forms possess a common dynamic core. In contrast, loop (β21–β24)_D2_ has enhanced mobility for holo-*Tth*-MCO and undergoes a conformational change that enables exposure to the proposed electron-transfer site (open conformation), while for apo-*Tth*-MCO, this loop prevents access to the electron-transfer site (close conformation), revealing the importance of good coordination among the copper ions and the histidine residues in the regulation of substrate binding. On the other hand, extended MD simulation of the open conformation with the electron-donor molecule docked into the protein cavity, showed that this conformation is required for the optimal electron transfer and that residues near the cavity play an important role in the process.

## Methods

Crystallographic structures of apo-*Tth*-MCO (PDB entry 2XUW) and holo-*Tth*-MCO (PDB entry 2XU9) were used for the initial coordinates of the MD simulations [13]. The qualities of the X-ray structures were corroborated with WHAT IF Web interface [18].

MD simulations were performed using GROMACS 4.0.5 software, with the GROMOS 53a6 force field [19]. In our models, basic residues are protonated and acidic residues are unprotonated. Non-coordinating histidine residues were set at neutral charge, Cu-coordinating histidine residues were protonated in ND1 or NE2, according to its coordination with the copper ions or according to the optimal H-bonding conformation for apo-*Tth*-MCO and holo-*Tth*-MCO. To keep the correct orientation between the histidines and the copper atoms in holo-*Tth*-MCO, copper atoms were coordinated to the polypeptide chain by constraining the distances from Cu to the histidine ligand atoms, to their crystallographic distances [13]. A strong charge transfer between the S atom of the Cys445 ligand and the T1Cu site, was modeled by setting a partial charge of +0.5 e on the T1Cu site and −0.5 e on the unprotonated thiol, as in other MD simulations of copper-containing proteins [20]–[24]. Both systems were neutralized and solvated in a periodic dodecahedric box containing a simple point charge (SPC) water model [25] that extended to at least 1 nm between the complex and the edge of the box, in a solution of 100 mM NaCl. All the simulated boxes contained about 17,000 water molecules. Simulations were carried out at a constant temperature of 300 K. Although holo-*Tth*-MCO is a hyperthermophile enzyme, simulations run at 300 K or 340 K with other hyperthemophylic systems have given similar results [26], and force fields are currently optimized and predominately used around 300 K [27], so 300 K was selected. Furthermore, holo-*Tth*-MCO is stable during simulation, and it has been observed in our research group that apo-*Tth*-MCO is unstable after exposing the enzyme in solution at room temperature by few hours, so 300 K appears to be sufficient for the comparison. However, in order to verify this assumption, a MD simulation of the holo-*Tth*-MCO was performed at 340 K for 36 ns.

Before every MD simulation, the internal constraints were relaxed by energy minimization, followed by 500 picoseconds (ps) equilibration under position restraints of the carbon backbone atoms through a harmonic forces constant of 5000 kJ/mol nm^2^. During the MD runs, covalent bonds in the protein were constrained using the LINCS algorithm [28]. The SETTLE algorithm was used to constrain the geometry of water molecules [29]. Berendsen’s coupling algorithm was used to maintain the simulation under constant pressure and temperature [30]. Van der Waals (VDW) forces were treated using a cutoff of 1.2 nm. Long-range electrostatic forces (r>1.2 nm) were treated using particle mesh implemented in the Ewald method [31]. Through the production runs, the trajectory data were saved every 1 ps, and the total duration of the simulations were 38 ns. The last configuration obtained from the simulation of holo-*Tth*-MCO was taken out and docked with ABTS to create a theoretical complex holo-*Tth*-MCO-ABTS. Starting with this configuration, a second MD simulation of the complex was carried out. After 1 ns equilibration the system ran for another 40 ns. Equilibration and simulation conditions were identical to those used for holo-*Tth*-MCO. The bonding parameters for ABTS were obtained from the Dundee PRODRG Server [32].

Root-mean-square deviations (RMSD) and root-mean-square fluctuations (RMSF) were calculated taking the energy-minimized structure as a reference. The radius of gyration (R_G_) and apolar and polar solvent accessible surface areas (SASA) were determined over all the protein atoms. Salt bridges (SB) were calculated for pairs of oppositely charged residues (Asp or Glu with Arg, His or Lys), whose side-chain charged groups were within a cutoff distance of 0.50 nm from each other. The number of intramolecular hydrogen bonds (HB_intra_) were calculated for all the trajectories using a donor-acceptor atom cutoff distance of 0.25 nm and a donor-hydrogen-acceptor angle of 135° [33]. The side-chains of charged groups involved in SB were discarded from the HB_intra_ analysis.

Principal components analysis (PCA) of the protein motion was determined from the diagonalization of the covariance matrix of the interatomic fluctuation [34], [35], providing a set of eigenvalues (amount of motion) associated to eigenvectors (direction of motion). The two extreme projections along the first eigenvector were calculated. Average conformations were calculated from the variance-covariance matrix of all protein atoms during the equilibrium time of the run.

Correlated atomic motions of the two systems were estimated by using dynamical cross-correlation motions (DCCM) [36]. DCCM may occur among neighboring residues that form a secondary structure, as well as among distant amino acids belonging to different regions or domains. DCCM indicate whether these residues move in the same (positive correlation) or opposite directions (negative correlation) [37]. For apo-*Tth*-MCO and holo-*Tth*-MCO the last 18 ns were taken as equilibrium time, whereas that for the holo-*Tth*-MCO-ABTS complex the last 25 ns were used for analysis, unless otherwise stated. Tools from the GROMACS package and a modified version of g_covar were used for the analysis of the data [19]. All graphical representations were generated by PYMOL version 0.99rc6 [38].

## Results and Discussion

### Stability

Geometrical properties such as the average values of the solvent-accessible surface area (SASA) both polar and apolar, the radius of gyration (R_G_), and the number of intra-protein hydrogen bonds (HB_intra_) were monitored as a function of time, to assess the simulation’s stability (Table 1). All of these properties indicate that the structures were stable during the entire simulation. The two *Tth*-MCO forms shared almost identical values for SASA and R_G_. However, the HB_intra_ reflects a slight difference between the two protein forms.

**Table 1:** Selected structural properties of *Tth*MCO apo and holo forms.

System	HB_intra_	R_G_ (nm)	Apolar SASA (nm^2^)	Polar SASA (nm^2^)
apo-*Tth*-MCO	193±2	2.13±0.01	188±3	130±2
holo-*Tth*-MCO	202±2	2.15±0.01	192±3	133±2

**Figure 2:**
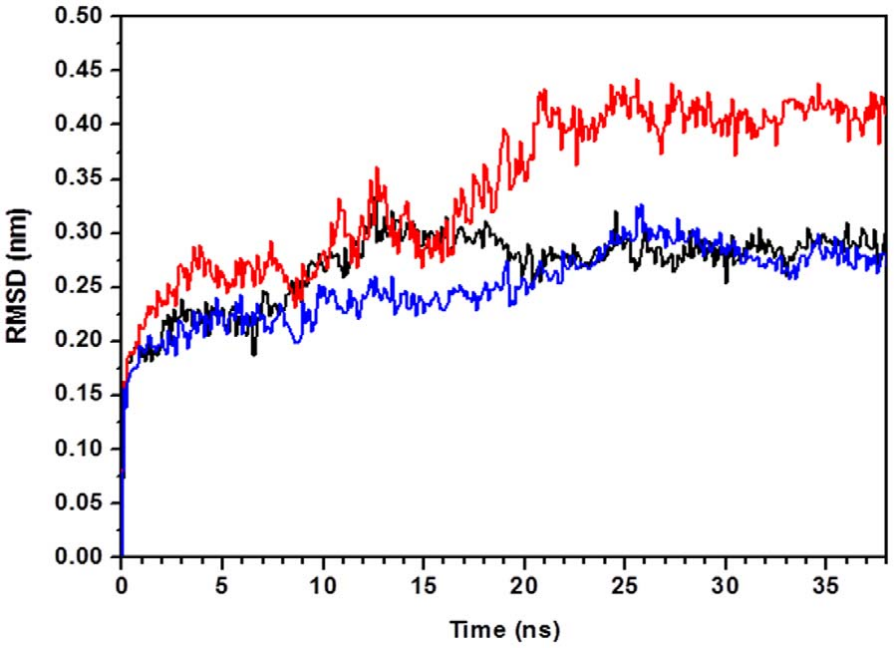
Root mean square deviation (RMSD) from the crystallographic structures of the C^α^ atoms as a function of simulation time for apo-*Tth*-MCO form (black line), holo-*Tth*-MCO (red line) and holo-*Tth*-MCO without loop (β21–β24)_D2_ (blue line).

More information about protein equilibration is provided by convergence of the root mean square deviation (RMSD) of the atomic position, with respect to the initial structure, calculated as a function of simulation time. In Fig. 2, the RMSD of the C^α^ atoms from the starting position are shown for apo-*Tth*-MCO (black line), holo-*Tth*-MCO (red line) and holo-*Tth*-MCO without loop (β21–β24)_D2_ (blue line). The comparison of these plots indicate that the apo-*Tth*-MCO form requires almost the same time to equilibrate with respect to holo-*Tth*-MCO without the loop (β21–β24)_D2_, while holo-*Tth*-MCO requires at least 20 ns to equilibrate. In general, the RMSD for apo-*Tth*-MCO and holo-*Tth*-MCO without the loop (β21–β24)_D2_ is lower (0.28±0.01 nm) than holo-*Tth*-MCO (0.41±0.013 nm) for the last 18 ns, the time where both forms are in equilibrium. From a structural point of view, the loop (β21–β24)_D2_ appears to be responsible for the greater time that holo-*Tth*-MCO takes to reach equilibrium. Furthermore, it is worth nothing that MD simulations of holo-*Tth*-MCO at 340 K showed a similar dynamic behavior for the loop (β21–β24)_D2_, but the final conformation (open conformation) was reached after 6 ns (fig. S1).

### Conformational Mobility of Loop (β21–β24)_D2_

RMSD analysis between the crystallographic apo-*Tth*-MCO and holo-*Tth*-MCO structures show a value of 0.014 nm, suggesting no significant conformational changes accompanied the copper coordination. Notwithstanding, the preference of this conformational state might be because the crystalline matrix of both forms is stabilized by contacts between a β-hairpin (residues 292 to 307) localized into the loop (β21–β24)_D2_ of symmetrically related copies [13]. In addition to this, structure-similarity searches using the program DaliLite [39] and considering *Tth*-MCO as a query, show that CueO (laccase from *Escherichia coli*, PDB entry 1N68) and CotA (MCO from *Bacillus subtilis*, PDB entry 1UVW), have the highest Z scores (34 and 26, respectively). Structural comparison of both MCOs with *Tth*-MCO were 0.12 nm (analysis performed on 233 C^α^ atoms) and 0.14 nm (analysis performed on 220 C^α^ atoms) for CueO and CotA, respectively. This analysis shows that the main structural differences among them are situated close to the T1Cu site (fig. 1C). Close to this place is where the electron-transfer site has been identified for the CotA-ABTS complex [40, PDB entry 1UVW] and therefore the likely electron-transfer site for holo-*Tth*-MCO. Furthermore, a recent study of MD simulations revealed that an increased mobility of the loops forming the reducing electron-transfer site may be consistent with increased activity of the POXA1b holo enzyme [41]. Another research group reported that deletion of the methionine-rich helical region covering the substrate binding site in CueO, increases its activity but decreases its specificity [42].

**Figure 3:**
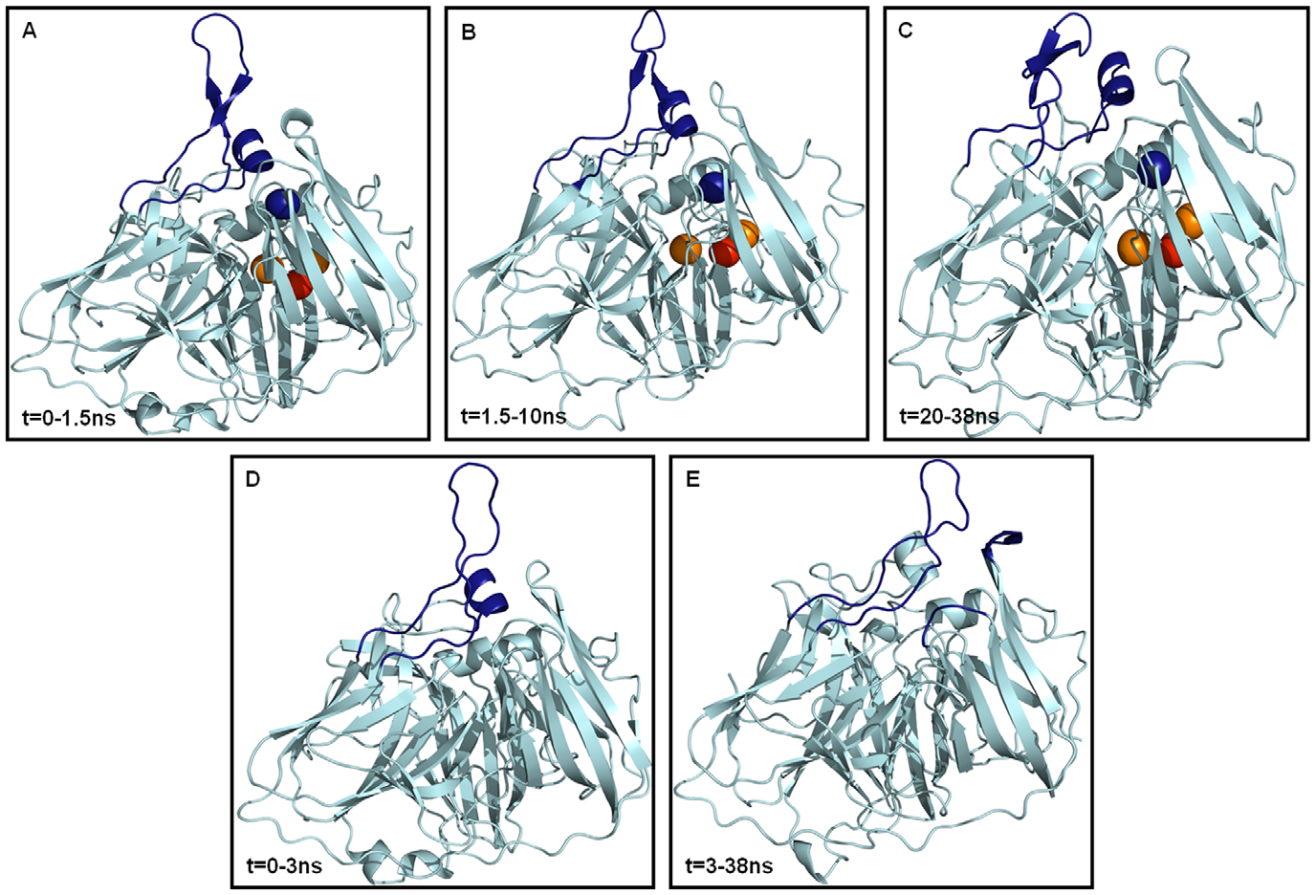
Structural evidences of the average conformations accessible in the 38 ns MD simulation for the holo-*Tth*-MCO (snapshots A–C) and apo-*Tth*-MCO forms (snapshots D–E). The different secondary structure elements are represented in light blue cartoons. The protein regions involved in the open-closure of the electron-transfer site are in deep blue cartoons: Loop (β21–β24)_D2_ and α4-helix_D2_ for snapshots A-B and D, loop (β21–β24)_D2_ and (187-α4-201)_D2_ for snapshot C, loop (β21–β24)_D2,_ loop (β25–β26)_D3_ and loop (β28–β29)_D3_ for snapshot E.

Snapshots A through C in Figure 3 illustrate average conformations in the 38 ns MD simulation for holo-*Tth*-MCO. Snapshot A shows the initial structure; this species was present within the first 1.5 ns of the simulation. During this time the loop (β21–β24)_D2_ was establishing interactions with the α-helix of the second domain (α4-helix_D2_, residues 190–197), which is located between β-strands 12 and 13 (residues 188–201) of the second domain (187-α4-201)_D2_). After this simulation time and until 10 ns, loop (β21–β24)_D2_ continued establishing interactions with the same α4-helix_D2_, but the interactions were tighter (fig. 3B). From 10 to 17 ns, loop (β21–β24)_D2_ made interactions with several residues from (187-α4-201)_D2_, which embrace the α4-helix_D2_, (no snapshot shown). From 20 to 38 ns, the protein conformation was almost the same as the latter conformation depicted above (fig. 3C), but the fluctuation was lower, mainly because of tighter interactions between loops (β21–β24)_D2_ and (187-α4-201)_D2_. Snapshots D through E in figure 3 show the two average conformations accessible in the 38 ns MD simulation for apo-*Tth*-MCO. Snapshot D shows the initial conformation for this species, which was present within the first 3 ns of the simulation time, when loop (β21–β24)_D2_ was establishing interactions with the α4-helix_D2_. After this simulation time and until the end of the MD simulation time (38 ns), loop (β21–β24)_D2_ was forming interactions with the loop between β-strands 25 and 26 of the third domain (loop (β25–β26)_D3_, residues 354–357) and with the loop between β-strands 28 and 29 of the third domain (loop (β28–β29)_D3_, residues 389–392) (fig. 3E). This latter conformation could be responsible for the slightly lower polar and apolar SASA values observed for this conformational state (table 1).

The analysis of contact numbers for both systems generally shows that internal contacts for the two forms are roughly the same (fig. S2). Nevertheless, because of the conformational preferences of loop (β21–β24)_D2_ in both species, the number and type of contacts between loop (β21–β24)_D2_ and the rest of the protein shows some differences. This analysis shows that apo-*Tth*-MCO establishes contacts within a short distance of loop (β25–β26)_D3_ and loop (β28–β29)_D3_, with an average contact number of 97±14. It is worth mentioning that all these regions are close to the substrate binding site. For holo-*Tth*-MCO, loop (β21–β24)_D2_ makes contact only with the loop (187-α4-201)_D2_ with an average contact number (62±15). These findings, coupled with structural evidence (fig. 3), point out that in spite of showing different conformational preferences between loop (β21–β24)_D2_ and the rest of the protein, both conformational states sought in the MD simulation reduce their volume. This explains why there are no significant differences in the R_G_ and SASA values for the two systems. On the other hand, this analysis has also shown that apo-*Tth-*MCO establishes a higher number of hydrogen bonds with loops (β25–β26)_D3_ and (β28–β29)_D3_ (9±2) than does holo*-Tth-*MCO (3±1), in contrast with the slightly higher HB_intra_ found for holo*-Tth-*MCO (table 1).

### Structural Analysis

**Figure 4:**
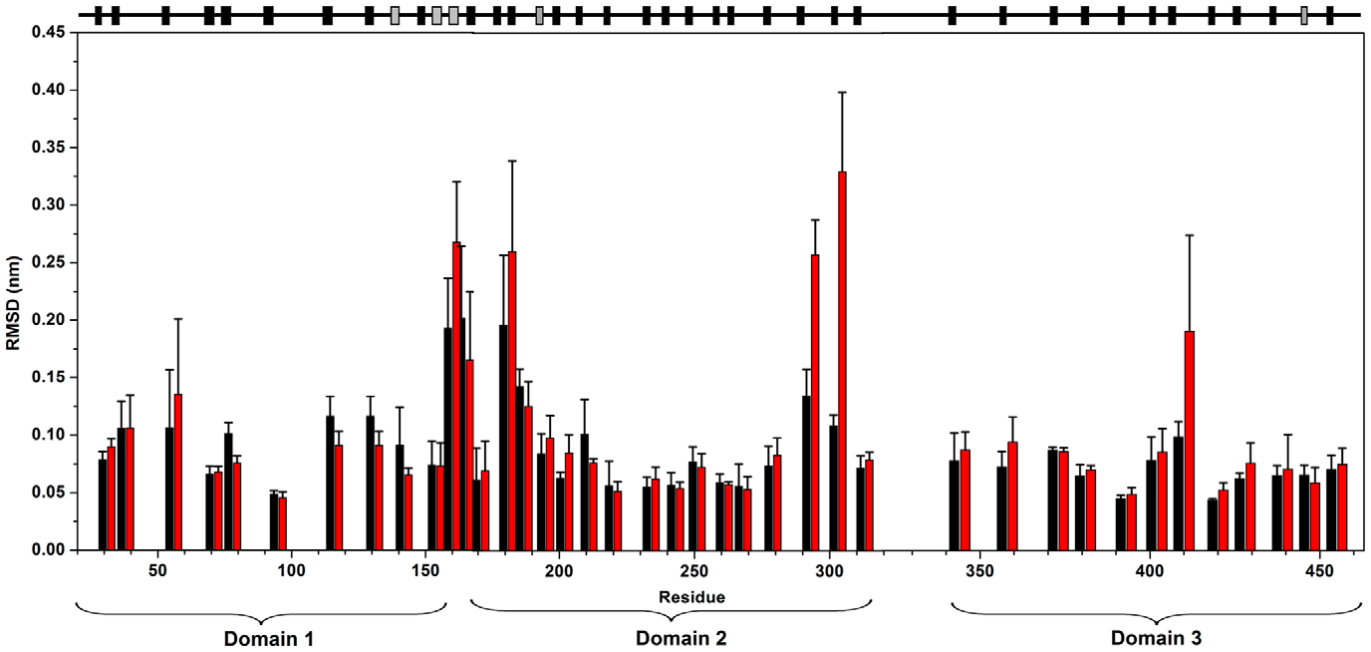
Root mean square deviations (RMSD) from the starting structure of the C^α^ atoms, as a function of the residue number, for apo-*Tth-*MCO (black) and holo-*Tth-*MCO (red) for the last 18 ns of a 38 ns-long simulation. Deviations are averaged over C^α^ fragments with a homogeneous secondary structure. Error bars represent the standard deviation. Secondary structure elements are shown at top: β-sheet (black) and α-helix (grey).

With the aim to investigate the conformational changes experienced for both protein forms in the MD simulation, the deviation of the C^α^ was computed as a function of the sequence index. In order to reduce the mobility of specific residues, the RMSD for the C^α^ was calculated and averaged over consecutive residues which shared the same secondary structure. The product of this analysis was 40 fragments, which included all the 35 protein β-strands, plus five α-helices. The result obtained in the interval time of 20 to 38 ns is reported in figure 4. It is evident that large atomic fluctuation is found for holo-*Tth*-MCO in correspondence with the loop (β21–β24)_D2_. In fact, high B-values in this region are also found for the X-ray structure [13]. In contrast, all the other regions show a similar structural behavior for the two protein species and are characterized by low atomic deviation, with RMSD values of 0.1 nm or lower. This is due to the fact that the overall structure is strictly conserved in both apo and holo forms. This outcome is in agreement with other MD simulation studies of copper-proteins [43].

The secondary structure behavior of both forms was calculated using the DSSP program [44] in the course of the MD simulation (fig. S3). It is evident at 38 ns of MD simulation that the protein structure, on the overall, is remarkably constant. A different behavior is observed in the first half of the trajectory, as compared to the second half of the trajectory, when the protein structure is more equilibrated. The structure of apo-*Tth*-MCO shows an α-helix structure in the region (α-helices 2 and 3, residues 158–168), which connects the first domain with the second domain (α2-α3-helix_D1–D2_), in 20 of 38 ns of the MD simulation (from 0–7 and 17–30 ns). For holo-*Tth*-MCO, this region is an α-helix for only the 2 first ns, after this time and until the end of the simulation it shows a structural behavior characteristic of a loop. On the other hand, the β-strand 24 of the second domain (residues 313–320) for holo-*Tth*-MCO shows a coiled structural behavior from 28 to 38 ns, while apo-*Tth*-MCO maintains the β-strand structure throughout the entire MD simulation time. This β-strand 24 is connected to β-strand 25 by a loop (residues 321–343) (fig. 4), which links the domain 2 and domain 3 (loop (β24–β25)_D2–D3_) and to the other side by loop (β21–β24)_D2_. In spite of the fact that these different structural behaviors suggest a higher mobility in this region for holo-*Tth*-MCO, the atomic deviation for both proteins is almost the same (fig. 4) Overall, it is clear that despite the high atomic deviation observed in loop (β21–β24)_ D2_ for holo-*Tth*-MCO form, the β-structure is preserved in both molecules (fig. 4).

Salt bridges can have an important stabilizing effect on protein structure, particularly when they are embedded in a hydrophobic environment such as the protein core [45]. In this work, only the pairs were considered when they were within a certain cut-off distance from each other along the entire MD simulation (<0.50 nm), a similar approach to that used in structural studies and MD simulations of proteins [45]–[47]. Table 2 shows the salt bridges present in the average conformation, as seen during the last 18 ns of time for MD simulation of both conformational states. The results are in agreement with the X-ray experiments: all the most frequent salt bridges present during the MD simulation exist in the crystal structure. The number of salt bridges is higher for holo-*Tth*-MCO. Furthermore, all of them are formed along the entire structure (table 2). On the other hand, structural analysis of the oppositely charged residues that established salt bridges in holo-*Tth*-MCO, but not in apo-*Tth*-MCO, revealed than most of them were forming hydrogen bonds. For holo-*Tth*-MCO the salt bridges:K43(NZ)-E85(OE2), E90(OE1)-R141(NH1), E171(OE1)-R223(NH2), H137(ND1)-E451(OE2), D178(OD1)-R231(NH1), E199(OE2)-R290(NH1), R290(NH2)-D392(OD1) and D367(OD1)-R369(NH2) were replaced by hydrogen bonds in apo-*Tth*-MCO:A46(N)-E85(O), E90(O)-N86(ND2), R223(NH2)-T131(OG1), E451(OE2)-H139(N), D178(OD1)-R231(N), R290(NH1)-D392(O), E199(OE2)-S310(N). However, in spite of this, the number of hydrogen bonds was higher for holo-*Tth*-MCO. These findings suggest that higher thermal stability could be expected in the holo form because of the improved energetic contribution of the hydrogen bonds and salt bridges. Furthermore, structural studies of thermophilic holo proteins have found a strong correlation between the number of salt bridges and protein thermal stability [48]–[51], in agreement with the high thermophilic behavior observed for holo-*Tth*-MCO [12].

**Table 2:** Salt bridges found for apo-*Tth*-MCO and holo-*Tth*-MCO.

Domain	Interaction	Apo-*Tth-*MCO	Holo-*Tth-*MCO
D1–D2*	E29(OE2)-H172(ND1	0.28	2.7
D1*	K43(NZ)-E85(OE2)	–	0.48
D1*	R73(NH2)-E157(OE2)	0.40	0.35
D1–D2*	R73(NH2)-E171(OE2)	0.40	0.44
D1*	R81(NH2)-E121(OE1)	0.43	0.47
D1*	E85(OE2)-R87(NH2)	0.41	0.48
D1*	E90(OE1)-R141(NH1)	–	0.39
D1–D3*	H137(ND1)-E451(OE2)	–	0.47
D1–D3*	H139(ND1)-E451(OE2)	3.2	0.40
D2*	E168(OE1)-K217(NZ)	–	0.47
D2*	E170(OE1)-H172(NE2)	0.37	0.37
D2^x^	E171(OE1)-R221(NH1)	0.42	0.45
D2^x^	E171(OE1)-R223(NH2)	–	0.42
D2^x^	D178(OD1)-R231(NH1)	–	0.46
D2–D3^x^	H189(NE2)-E449(OE1)	0.27	0.32
D2*	D193(OD2)-K198(NZ)	0.33	0.30
D2*	E199(OE2)-R231(NH2)	0.48	0.44
D2*	E199(OE2)-R290(NH1)	–	0.41
D2*	R210(NH2)-E313(OE1)	0.44	–
D2^x^	R221(NH2)-E270(OE2)	0.32	0.32
D2^x^	R223(NH2)-E270(OE1)	0.41	0.42
D2*	R234(NH2)-E260(OE1)	0.34	0.38
D2^x^	D248(OD2)-R268(NH1)	0.42	0.36
D2–D3^x^	D248(OD2)-K418(NZ)	0.32	0.35
D2–D3^x^	E267(OE2)-K418(NZ)	0.32	0.36
D2–D3^x^	R268(NH2)-D419(OD1)	0.32	0.37
D2–D3*	R290(NH2)-D392(OD1)	–	0.48
D3*	R346(NH2)-E381(OE1)	0.38	0.36
D3*	R347(NH2)-E384(OE1)	0.45	0.48
D3*	D367(OD1)-R369(NH2)	–	0.47
D3*	H368(ND1)-D372(OD1)	–	0.29
D3*	K374(NZ)-E460(OE1)	0.47	0.48
D3^x^	E384(OE2)-R430(NH2)	0.43	0.43
D3^x^	D392(OD2)-K424(NZ)	–	0.35
D3*	H400(ND1)-E437(OE2)	0.37	0.27
D3*	R440(NH2)-E460(OE2)	0.43	–

Salt bridges present in the average conformation within a cutoff distance of 0.50 nm from each other, as seen during the last 18 ns of time for MD simulation of both conformational states (see methods). D1, salt bridge in domain 1; D2, salt bridge in domain 2; D3, salt bridge in domain 3; D1–D2, salt bridge between domains 1 and 2; D1–D3, salt bridge between domains 1 and 3; D2–D3, salt bridge between domains 2 and 3. Salt bridge *solvent-exposed,^x^solvent buried and larger than 0.5 nm (–).

### Flexibility

**Figure 5:**
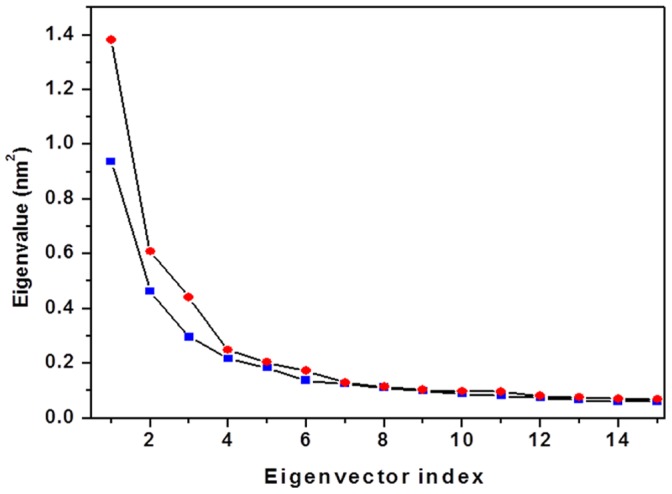
Eigenvalues as a function of the first 15 eigenvectors, in the time interval 20–38 ns, for apo-*Tth*-MCO (blue solid square) and holo-*Tth*-MCO (red solid circles).

The principal components analysis (PCA) [34], [35] was used to dissect out cooperative inner motions, to get further insights into the dynamics of both conformational states. For both systems, analysis of the trajectories was performed over the last 18 ns of simulation time, when the protein structure is sufficiently equilibrated. Only the C^α^ atoms were considered, and the starting structure was used as a reference structure in order to remove the roto-translation of the protein backbone in the simulation. Figure 5 shows the eigenvalues (mean square positional fluctuations) of the covariance matrix as a function of the first eigenvectores (degrees of freedom). Fluctuations are described completely by 15 eigenvectors in both systems (fig. 5). However, a higher amount of motion is experienced along the first two eigenvectors by holo-*Tth*-MCO (1.38 and 0.60 nm^2^), with respect to apo-*Tth*-MCO (0.94 and 0.46 nm^2^), which reflects the differences in the dynamic behavior of the two molecules. In fact, 70% and 65% of the total conformational fluctuations for holo-*Tth*-MCO and apo-*Tth*-MCO is concentrated in their first fifteen eigenvectors, respectively. This result is different compared to other proteins, since usually 10–20 eigenvectors correspond to 90% of the conformational fluctuations [34], [52]. However, this dissimilarity can be explained in terms of the rigid body-like behavior of *Tth*-MCO, a particularity that is shared with azurin and other cupredoxins [52].

**Figure 6:**
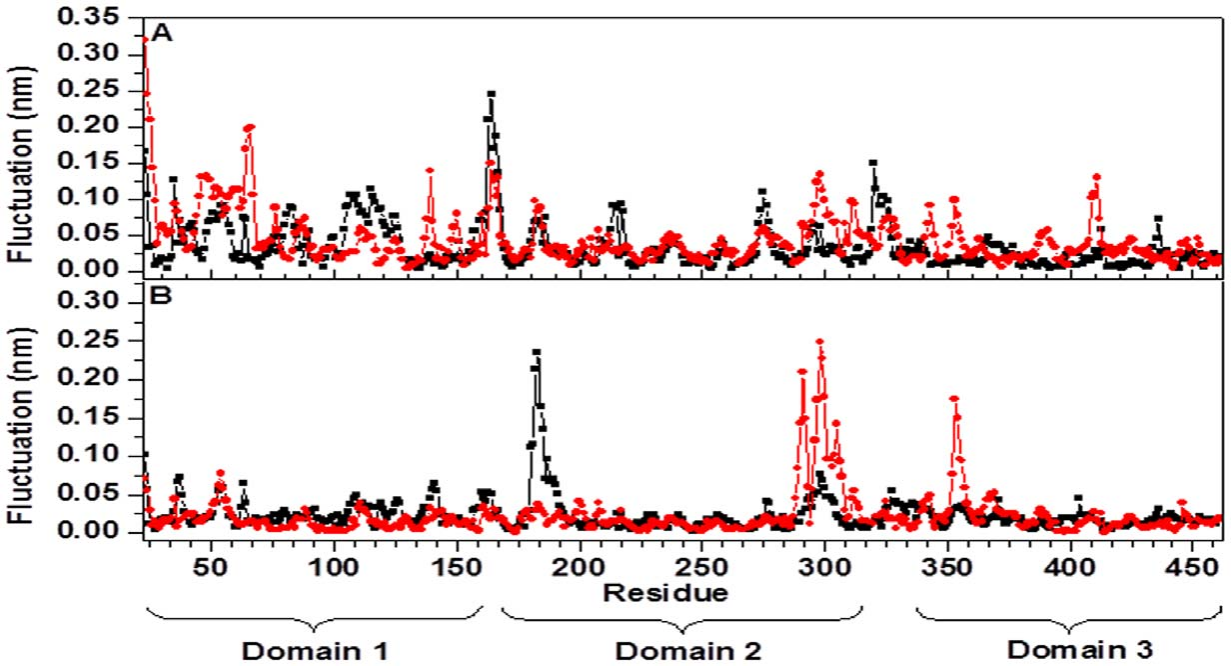
Fluctuations of the C^α^ atoms in the first (A) and the second (B) eigenvector for the time interval 20–38 ns, as a function of the residue number, for apo*-Tth-*MCO form (square black symbol) and holo-*Tth*-MCO form (circle red symbol).

The difference in the curves in figure 5 can be explained by investigating the contribution of each residue to the first few, and most relevant, eigenvectors. In figure 6 the fluctuations relative to the first (fig. 6A) and second (fig. 6B) eigenvector are reported as a function of the residue number. It is evident from the curves that the major motions captured by the first eigenvector are loops (β2–β3)_D1_, (β3–β4)_D1_ for holo-*Tth*-MCO and the α2-α3-helix_D1–D2_ for apo-*Tth*-MCO, whereas that the most important motions caught by the second eigenvector are loops (β21–β24)_D2_ and (β25–β26)_D3_ for holo-*Tth*-MCO, and the loop (β11–β12)_D2_ for the apo-*Tth*-MCO. The higher amount of motions observed for the second eigenvector in holo-*Tth*-MCO might be related to the electron-donor molecule interaction, while the first eigenvector could be associated with the O_2_ channel generation. On the other hand, the motions experienced by the first and second eigenvectors in apo-*Tth*-MCO are possibly related with the closure of loop (β21–β24)_D2_.

### Dynamical Cross-correlation Motions (DCCM)

**Figure 7:**
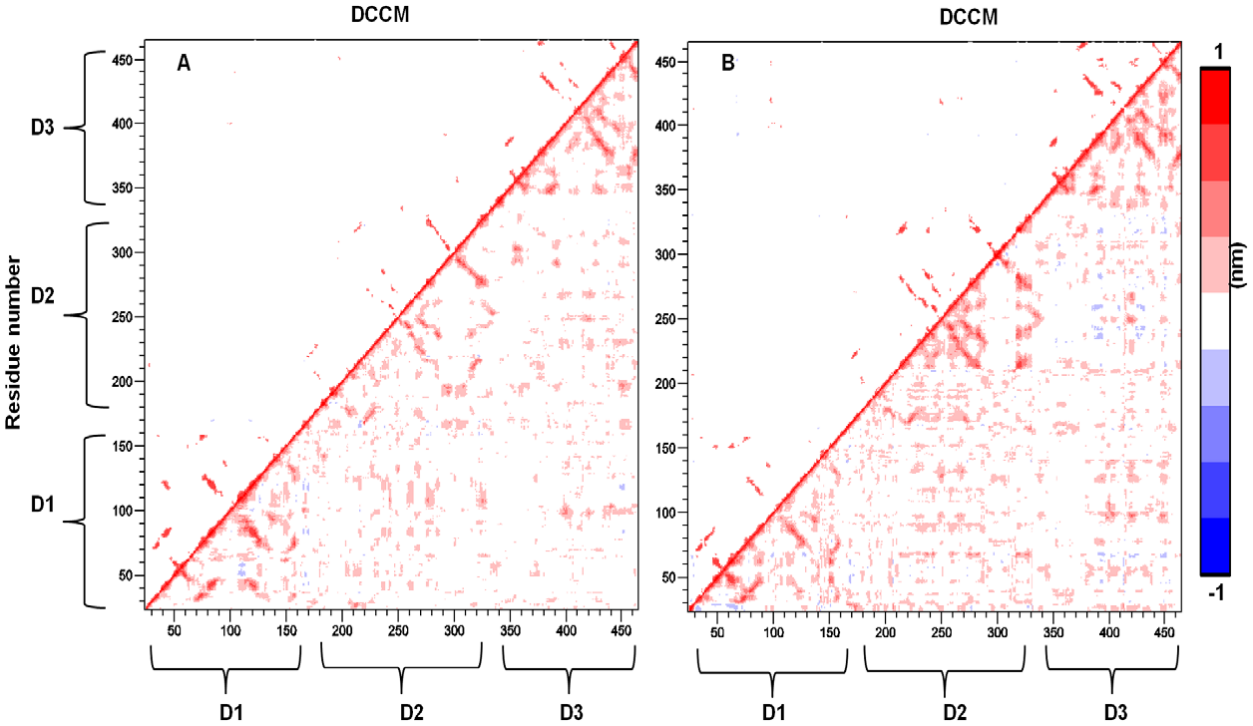
Cross-correlation motions (DCCM) for apo-*Tth-*MCO form (panel A) and holo-*Tth*-MCO (panel B). DCCM larger than 0.5 nm are shown on the upper triangle and all values on the lower triangle. Vector products representing the maxim extent of correlated motion (nm) for each C^α^ pair are plotted. The color scale indicates the degree of correlation: red, positively correlated; blue, negatively correlated; white, uncorrelated.

To further investigate how domain-domain communication affects the structural dynamics of loop (β21–β24)_D2_, DCCM representing the correlated motions were averaged over the last 8 ns of the MD simulation using 50 ps block length and is shown in fig. 7. The cross-correlation map averaged over the last 8 ns was found to agree with results obtained over the time interval of 20–30 ns, indicating that a converged picture of the correlated motions is obtained. From figure 7 it is possible to observe that for both species, most of the correlated motion corresponds to positive correlation values between intradomain and interdomain regions (fig. 7, red regions), whereas only a few negative values are observed (fig. 7, blue regions). All these cross-correlations are explained by bonded and non-bonded interactions established in each cupredoxin domain and among them as well (table S1). In general, for holo-*Tth*-MCO a stronger degree of correlation is found throughout the map within the second domain, between the second domain, α2-α3-helix_D1–D2_ and loop (β24–β25)_D2–D3_, and between loop (β21–β24)_D2_ and loop (β24–β25)_D2–D3_, whereas for apo-*Tth*-MCO many of these correlations are not observed. This marked qualitative difference in the correlation pattern further confirms that the conformational dynamic of loop (β24–β25)_D2–D3_ is caused by tight mechanical interdomain packing, enabling cooperation of the three domains in the copper coordination. On the other hand, the stronger cross-correlations found for holo-*Tth*-MCO might be due to a higher number of hydrogen bonds and salt-bridges found for this conformational state (table 1 and 2).

### Structural Dynamics of TNC

Examinations of several crystallographic structures of MCOs show that TNC is stabilized by a second sphere of residues with carboxylates such as Asp or Glu. These residues have been shown to play a role in the structural integrity of the TNC of MCOs and it appears that some of them might contribute with a proton in the reduction of O_2_ to H_2_O [53], [54]. Structural analysis of both conformational states of *Tth*-MCO show that the second sphere of residues with carboxylate is constituted by Asp106, Asp419 and Glu451. The distance between the two aspartic acid residues and the TNC is ∼0.4 nm, while the distance between Glu451 and the TNC is ∼0.6 nm. Furthermore, theoretical calculation of pKa for Glu451 reveals that it may contribute a proton, because of its anomalous pKa = 9.9 (calculated with PDB2PQR) [55]. Since these residues play an important role in the stability of TNC, we analyzed their conformational mobility in both conformational states.

**Figure 8:**
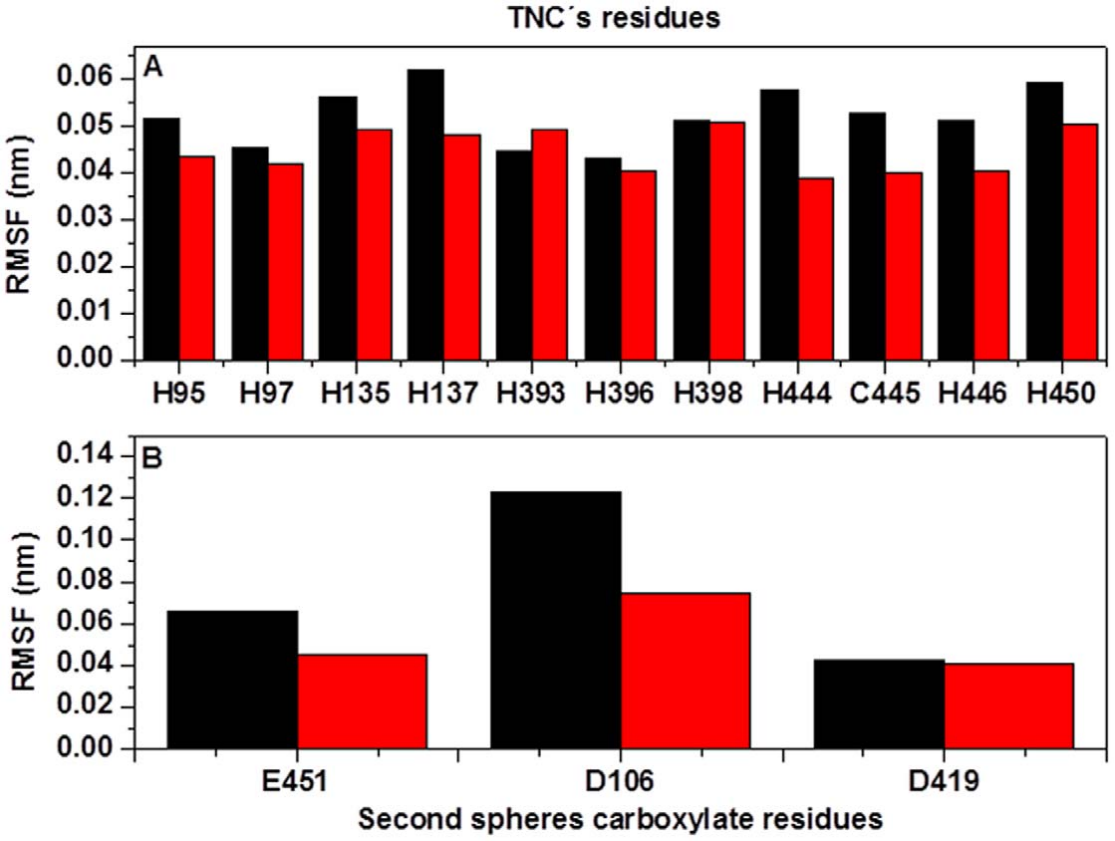
RMSF analysis of the C^α^ atoms of TNC’s residues (A) and the second sphere carboxylate residues (B), for apo-*Tth-*MCO (black) and holo-*Tth*-MCO (red) for the last 18 ns of a 38 ns-long simulation.

Figure 8A shows the comparative root mean square fluctuation (RMSF) analysis of the histidine residues and the acidic residues that stabilized TNC. These residues show a slightly higher mobility for apo-*Tth*-MCO. Moreover, this finding reveals that TNC is highly stable without cooper ions. Glu451 and Asp419 show similar mobility, while Asp106 has a higher mobility in apo-*Tth*-MCO (fig. 8B). The trajectory analysis shows that Asp106 of apo-*Tth*-MCO loses its coordination with TNC at the first 2 ns of MD simulation, causing a higher mobility in loop (β6–β7)_D1_ (fig. 6A). Asp106 is connected on one side by His97 and on the other side by His135, which in turn coordinates with a T3Cu. The higher mobility of these histidine residues in the apo-*Tth*-MCO causes Asp106 loses its coordination with TNC, contact that is observed in the initial structure [13].

Despite the fact that Asp419 shows the same mobility in both enzyme forms (fig. 8B), analysis of MD simulation trajectories shows that for holo-*Tth*-MCO, Asp419 establishes a good coordination with TNC (∼0.35 nm) and a salt bridge with Arg268 (see table 2), along the all MD simulation. On the other hand, apo-*Tth*-MCO shows poor interaction with neighboring residues of the former TNC and establishes a tighter salt bridge with Arg268 (see table 2). Contrary to what one would expect, the double interactions of Asp419 observed for holo-*Tth*-MCO increase mobility in loop (β30–β31)_D3_ instead of decreasing it (fig. 6A).

Glu451 has slightly less mobility in holo-*Tth*-MCO. This is because in holo-*Tth*-MCO, Glu451 establishes salt bridges with His 139 and His137 (table 2), while in apo-*Tth*-MCO, Glu451 makes interactions with His139 along the entire MD simulation (table 2). These findings point out that good coordination between cooper ions and histidine residues is essential, so that the acidic residues which stabilize TNC do not lose their coordination, as this fine conformational mobility might be involved in O_2_ uptake and the delivery of water.

### Simulation of the Holo-*Tth*-MCO-ABTS Electron-transfer Complex

As mentioned above, structural analysis of the crystallographic structures show that loop (β21–β24)_D2_ has the same conformation in both crystal structures, and forms a flexible lid at the entrance of the electron-transfer cavity which seems to occlude access to the electron-transfer site. On the other hand, MD simulation developed in this study, provide evidence that the loop (β21–β24)_D2_, experiences a conformational change on a timescale of 38 ns, which allows the exposition of the electron-transfer site. To confirm our hypothesis that the open conformation is required for the complex formation (holo-*Tth*-MCO-ABTS), the simulation was extended another 40 ns, but with the ABTS docked into the cavity of holo-*Tth*-MCO (see methods).

**Figure 9:**
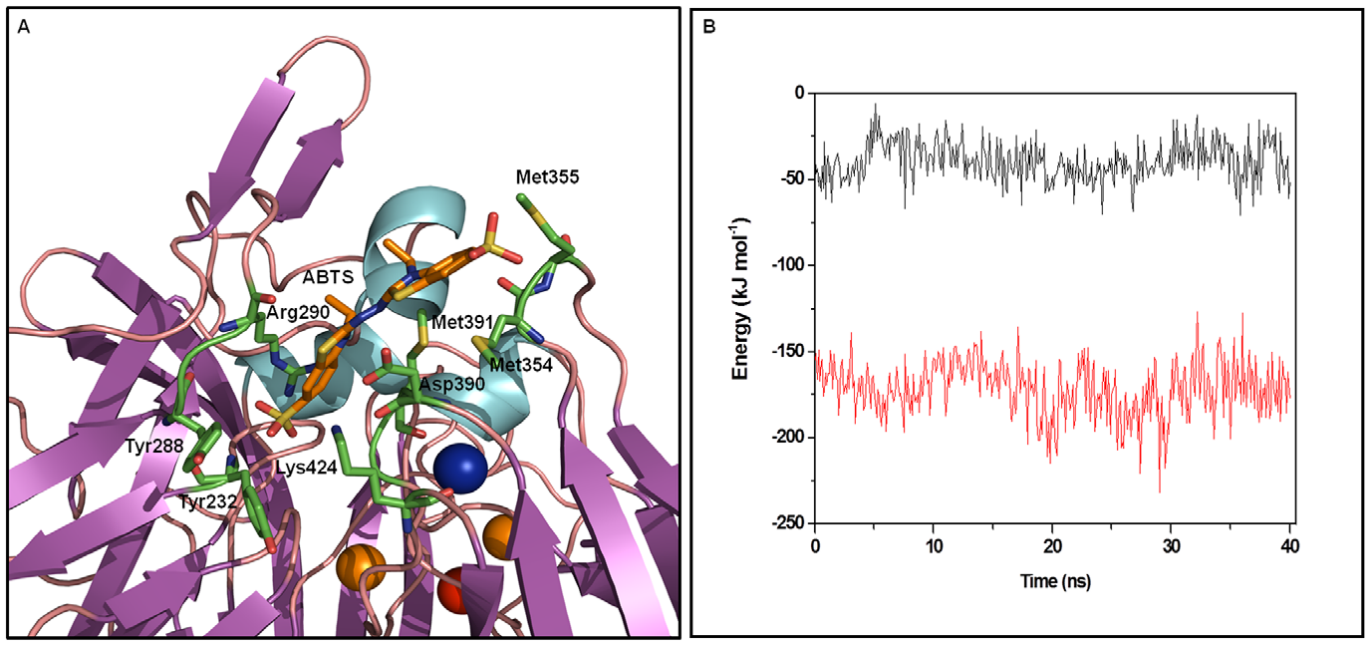
Average conformations of ABTS inside the electron-transfer complex formed with holo-*Tth*-MCO electron-transfer cavity during the last 25 ns of MD simulation. Residues in close proximity (<0.4 nm) to the ABTS (orange stick models) are represented as green stick models and coppers are shown in Van der Waals representation. B) The non-bonded short-range Coulomb (black line) and Lennard–Jones (red line) interaction between holo-*Tth*-MCO and ABTS as a function of time.

Structural analysis of the 40 ns MD simulation of holo-*Tth*-MCO-ABTS shows that after the energy minimization, ABTS is shown as a shallow conformation, U-shaped with the bottom of the U looking into the large pocket formed by apolar residues (fig. S4A). After 1 ns equilibration, the ABTS is shown with an almost linear shape, in which one-half of the adduct is partially buried, while the other half is relatively exposed to the external environment (fig. S4B). In the partially-buried half, ABTS is stabilized by Tyr232, Tyr288 and Lys424, while the exposed half establishes interactions with Met354, Met355 and Met391 and the middle part for Arg290 and Asp390 (fig. 9A). From the interval time of 1 to 7 ns of simulation, two conformations of the ABTS into de substrate cavity were present. The first one was mentioned above (fig. S4B), and the second one (fig. S4C) is where one part of the substrate was partially buried and stabilized by Tyr232, Tyr288 and Lys424 (as observed for the first conformation), while the other part was exposed to the solvent but did not establish any interaction. However, after this time and until the end of the 40 ns simulation, only the first conformation was present (fig. S4B).

RMSF analysis of the equilibrium time of the holo-*Tth*-MCO-ABTS complex (see methods), shows that when the substrate binds into the cavity, loops (β21–β24)_D2_ and (β25–β26)_D3_ experience a structural rigidification (fig. S5). This conformational reduction might contribute with a favourable enthalpic term for the complex formation, as observed for the complex formation of other systems through MD simulation methods [56], [57].

The position of ABTS in the substrate binding site of holo-*Tth*-MCO is in agreement with that observed for CotA-ABTS, where one-half of ABTS is buried and the other half is solved exposed [40]. Furthermore, the ligand geometry into the substrate binding site in both complexes might favor the transfer of an electron from the electron-donor molecule to the T1Cu [40]. However, the number of residues stabilizing the electron-transfer complex holo-*Tth*-MCO-ABTS is higher than that found in CotA-ABTS. In addition, it is worth nothing that in the complex CotA-ABTS, the authors mention that it was difficult to define the precise details of the adduct binding at a resolution of 2.4 Å [40], and even a careful evaluation of the electron density maps provided in the two CotA-ABTS complexes deposited at the PDB (PDB entries 1OF0 and 1UVW) allowed to have doubts about the occupation of the ABTS moiety modeled in the crystal (Adam Campos-Acevedo, unpublished results). On the other hand, it is important to notice that although ABTS is not a natural substrate for this enzyme, ABTS is oxidized, albeit at low rates, validating the model (Kevin Mendez-Acevedo and Brenda Valderrama, unpublished data).

In an attempt to trace the driving force behind interactions between holo-*Tth*-MCO and the ABTS, the non-bonding energy of ABTS interaction with the rest of the protein has been plotted as a function of time. From fig. 9B, it can be seen that the system is mostly stabilized by favorable Lennard-Jones (LJ) interactions, whereas the electrostatic contributions, although favorable, contribute in a smaller proportion to the complex stabilization. Despite the lower contribution of the electrostatic interaction, their role significant is setting up hydrogen bonds between the ligand and the complex (fig. 9A). Nevertheless, it is clear that hydrophobic effects are the main driving forces in this conformational state.

### Conclusion

MD simulations have been used to study the dynamic behavior of apo-*Tth*-MCO and holo-*Tth*-MCO, to explore the structural dynamics of loop (β21–β24)_D2_, as well as to compare basic features of the structure and dynamics, and how copper coordination affects the protein matrix. Our results show that apo-*Tth*-MCO equilibrates as fast as holo-*Tth*-MCO and shows similar atomic deviation values, when the loop (β21–β24)_D2_ is discarded from the RMSD analysis. They show similar SASA and R_G_ values, but differ in HB_intra_ and salt bridges. Conformational analysis of the average structures accessible in the 38 ns MD simulation showed that loop (β21–β24)_D2_ has enhanced mobility in holo-*Tth*-MCO and undergoes a conformational change that enables exposure to the electron-transfer site (open conformation), while for apo-*Tth*-MCO, this loop prevents access to the electron-transfer site (close conformation). At the same time, holo-*Tth*-MCO possesses a more flexible structure. This is evident in the PCA analysis; a significantly higher amount of motion is experienced along the second eigenvectors by holo-*Tth*-MCO. These motions are localized on loop (β21–β24)_D2_, and loop (β25–β26)_D3_, which are in close vicinity to the electron-transfer site. Noticeably, this nanosecond mobility of residues localized in the region surrounding the active site of holo-*Tth*-MCO, has been detected by azurin in different NMR experiments [58], [59].

Of particular interest are the results of domain communication, obtained from DCCM analysis. These shed light on the mechanical basis for interaction and reveal that mobility of loop (β21–β24)_D2_ is connected to copper coordination. The present result supports the hypothesis that copper coordination plays an important role in the overall motion of the protein scaffold. Furthermore, according to this picture, the greater flexibility of holo-*Tth*-MCO might facilitate its adaptation to greater electron-donor molecules selectivity. The difference in flexibility is coupled with a difference in the direction of internal motions, caused by coordination of copper to the enzyme.

On the other hand, the structural analysis of residues surrounding TNC pointed out that it is more rigid for holo-*Tth*-MCO, establishing interactions with Asp106, Asp419 and Glu451, in agreement with what is observed in the crystal structure. For apo-*Tth*-MCO, the interactions between these neighboring residues of the former TNC are lost because of a more relaxing mobility. These findings suggest that a good coordination of the copper ions and the histidines that build TNC is essential to maintain the second shell of acidic residues which stabilize the TNC.

Finally, MD simulation of the electron-transfer complex holo-*Tth*-MCO-ABTS confirmed our hypothesis that the open conformation favored the transient electron-transfer complex formation. Furthermore, this analysis pointed out that residues in loop (β21–β24)_D2_, Tyr288 and Arg290 seems to play an important role in stabilizing the complex, in which hydrophobic effects also might have an important role in driving this conformational change. Additionally, Met368 and Asp369 which participate in the stabilization of the electron-donor molecule into the electron-transfer site, are followed in sequence by His391 which coordinates T1Cu, being these residues potentially involved in the electron transfer to theT1Cu.

## Supporting Information

Figure S1 **Root mean square deviation (RMSD) from the crystallographic structures of the C^α^ atoms as a function of simulation time for holo-** ***Tth*** **-MCO (black line) and holo-** ***Tth*** **-MCO at 340 K (red line).**
(TIF)

Figure S2 **Inter-residue contact maps of** ***Tth*** **-MCO, as obtained from MD simulation (A) apo-** ***Tth*** **-MCO and (B) holo-** ***Tth*** **-MCO, for the last 18 ns of a 38 ns-long simulation.**
(TIF)

Figure S3 **Secondary structure of** ***Tth*** **-MCO as obtained from MD simulation (A) apo-** ***Tth*** **-MCO and (B) holo-** ***Tth*** **-MCO. Secondary structure elements are shown at bottom.** Figure calculated with DSSP [44].
(TIF)

Figure S4 **Holo-** ***Tth*** **-MCO-ABTS interaction modes.** A) Holo-*Tth-*MCO-ABTS conformation after the energy minimization. Two ABTS conformations present into the holo-*Tth*-MCO cavity during the first 7 ns of MD simulation (B) and (C).
(TIF)

Figure S5 **Fluctuations of the Cα atoms in the first (A) and the second (B) eigenvector for the time interval 20–38 ns, as a function of the residue number, for holo-** ***Tth-*** **MCO form (circle red symbol) and holo-** ***Tth*** **-MCO-ABTS electron-transfer complex (square black symbol).**
(TIF)

Table S1 **Correlated motions found in the apo and holo form of** ***Tth*** **-MCO and corresponding bonded or non-bonded interactions.**
(PDF)
