# Supplementary figures and images for: Molecular Dynamics of a Thermostable Multicopper Oxidase from Thermus thermophilus HB27: Structural Differences between the Apo and Holo Forms
Source: PLoS One. 2012 Jul 10;7(7):e40700. doi: 10.1371/journal.pone.0040700 (PMC3393687; doi:10.1371/journal.pone.0040700)

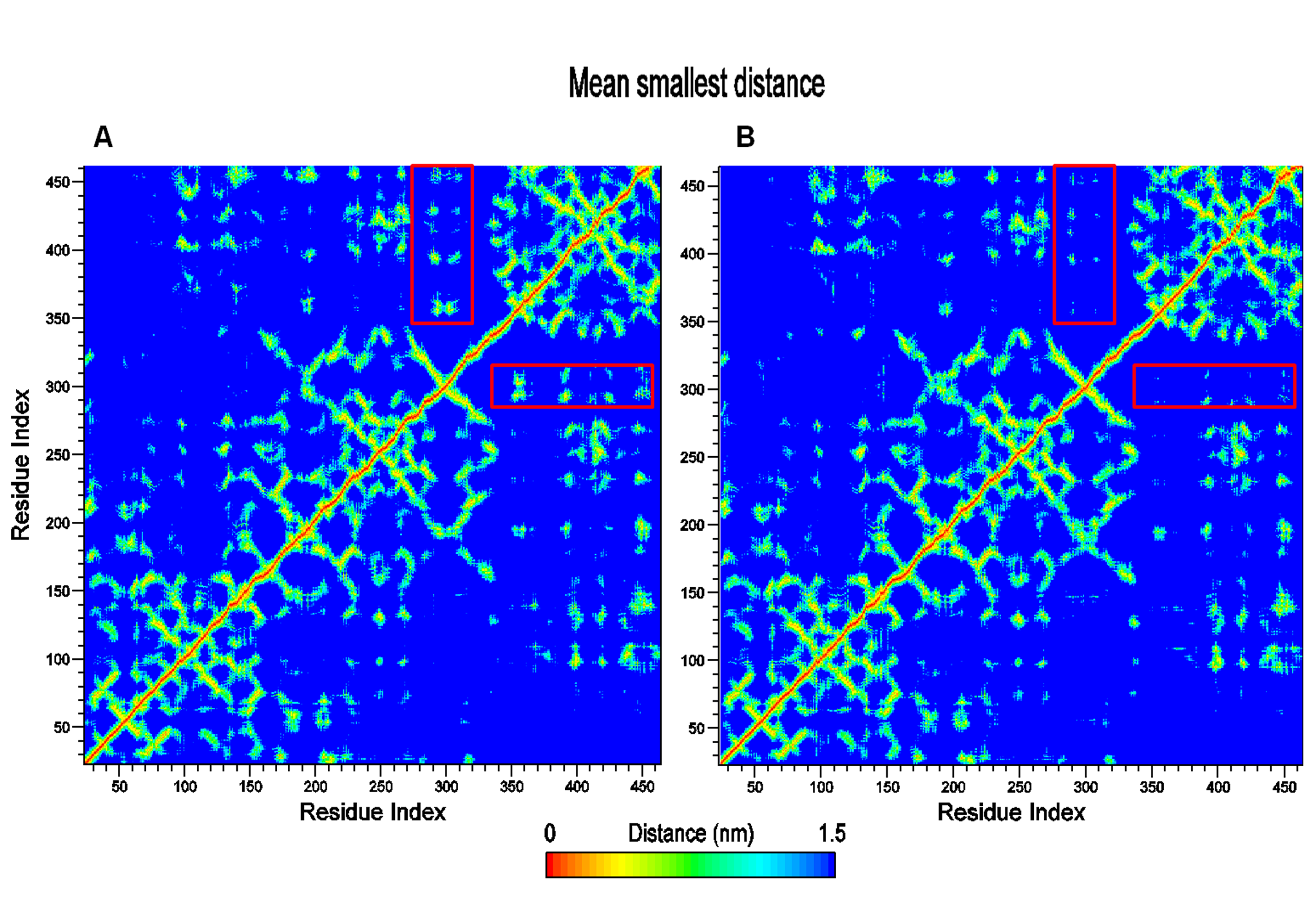

Supplement: Figure S2 — Inter-residue contact maps of Tth -MCO, as obtained from MD simulation (A) apo- Tth -MCO and (B) holo- Tth -MCO, for the last 18 ns of a 38 ns-long simulation. (TIF) [file pone.0040700.s002.tif]

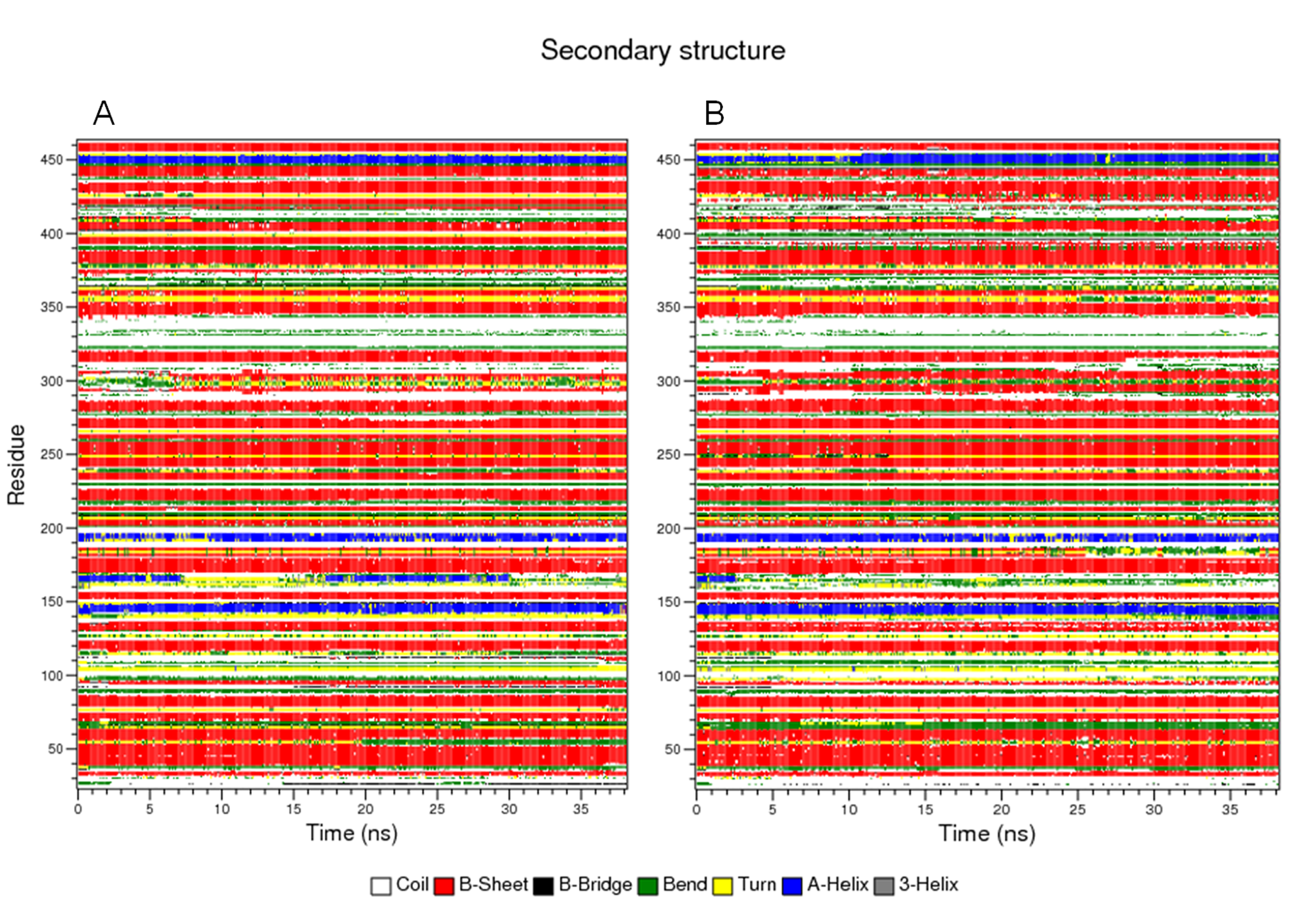

Supplement: Figure S3 — Secondary structure of Tth -MCO as obtained from MD simulation (A) apo- Tth -MCO and (B) holo- Tth -MCO. Secondary structure elements are shown at bottom. Figure calculated with DSSP [44]. (TIF) [file pone.0040700.s003.tif]

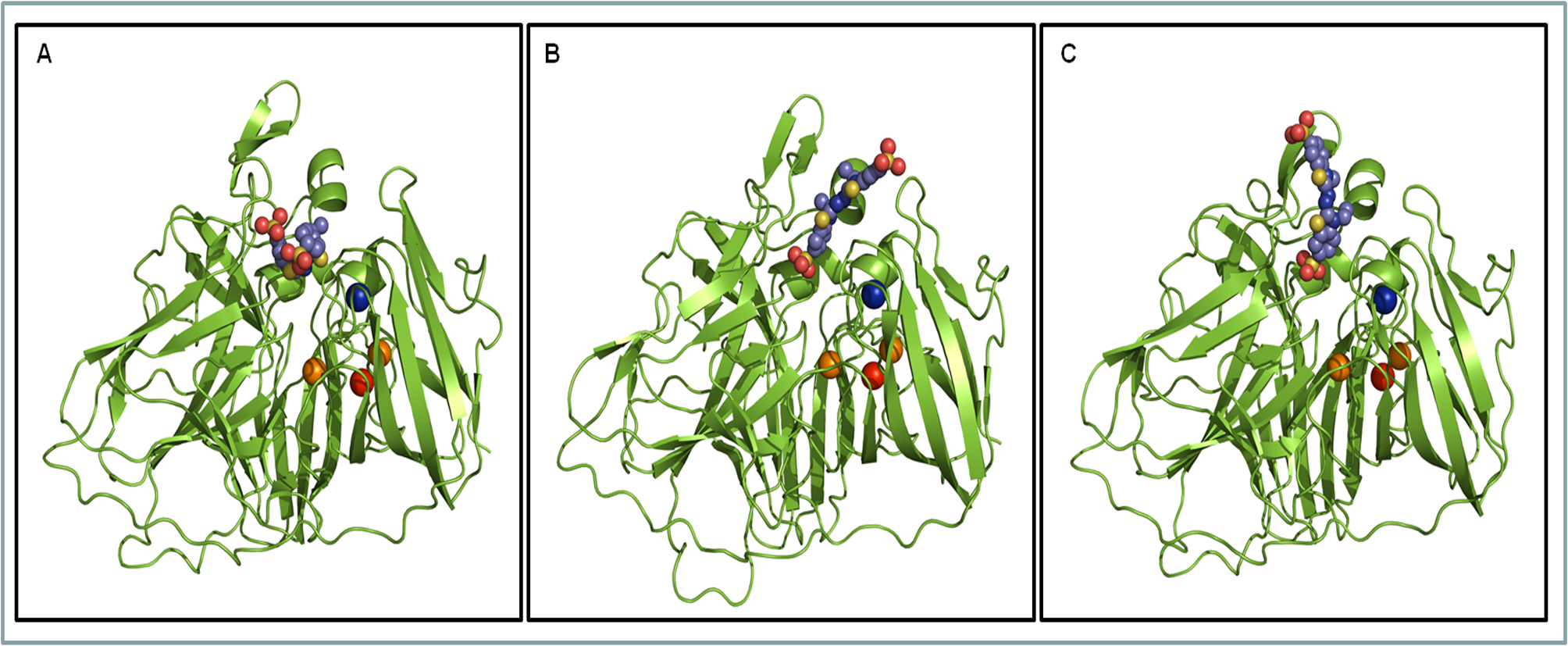

Supplement: Figure S4 — Holo- Tth -MCO-ABTS interaction modes. A) Holo-Tth-MCO-ABTS conformation after the energy minimization. Two ABTS conformations present into the holo-Tth-MCO cavity during the first 7 ns of MD simulation (B) and (C). (TIF) [file pone.0040700.s004.tif]
