# Supplementary material for: Molecular Dynamics of a Thermostable Multicopper Oxidase from Thermus thermophilus HB27: Structural Differences between the Apo and Holo Forms
Source: PLoS One. 2012 Jul 10;7(7):e40700. doi: 10.1371/journal.pone.0040700 (PMC3393687; doi:10.1371/journal.pone.0040700)
Supplement: Table S1 — Correlated motions found in the apo and holo form of Tth -MCO and corresponding bonded or non-bonded interactions. (PDF) [file pone.0040700.s006.pdf]

Table S1. Correlated motions found in the apo and holo form of *Tth*-MCO and corresponding bonded or non-bonded interactions.

Secondary structure	Interactions
Domain 1	
loop (β1-β2)/loop (β4-β5)[a]	HB:D78(sc)-S35(sc)-G37(bb), R75(sc)-Q36(sc)
loop (β3-β4)/loop (α1- β9)[a]	VdW:L151(bb)-F67(sc), HB:L151(bb)-G69(bb)
β1/loop (β5-β6)[a]	SB:K43(sc)-E85(sc), HB:E85(bb)-S45(bb)-A46(bb)
β4/(α1)[a]	SB: R73(sc)-E157(sc), HB:R73(bb)-E157(bb)
β5/β7[a]	SB:R81(sc)-E121(sc), HB:W118(bb)-Leu84(bb), Y120(bb)-L82(bb), T119(bb)-T83(bb)
loop (β5-β6)/ loop (β8-α1)[a]	SB:E90(sc)-R141(sc), HB:L138(bb)-T92(bb), H137(bb)-N93(bb)
α2-α3-helix$_{D1-D2}$ /(loop(β24-β25)$_{D2-D3}$[b]	VdW:L327(sc)-E168(bb), L160(sc)-L333(bb), I163(sc)-L333(bb), P164(sc)-P330(sc), HB:E165(sc)-K331(bb).
Domain 2	
β10/ β15[a]	SB:E171(sc)-R221(sc), E171(sc)-R223(sc), HB:T219(bb)-E170(bb), L176(bb)-L225(bb), R221(bb)-H172(bb), R223(bb)-H172(bb), VdW:L224(sc)-L174(sc), L222(sc)-H172(bb)
β10/ loop (β15-β16)[b]	SB: D178(sc)-R231(sc), 1VdW:L176(bb)-A227(bb), HB:S228(sc)-D178(bb)
β17/(loop(β24-β25)$_{D2-D3}$[b]	VdW:L329(sc)-T243(sc), L333(sc)-I245(sc)
β15/ β20 [b]	SB: R221(sc)-E270(sc), R223(sc)-E270(sc),VdW:L225(bb)-R268(bb), HB:L224(bb)-A269(bb), L222(bb)-V271(bb), L220(bb)-V273(bb)
β16/ loop (β18-β19)[b]	SB:R234(sc)-E260(sc)
loop (β20-β21) /(loop(β24-β25)$_{D2-D3}$[b]	VdW:P324(sc)-L275(bb), HB:E278(sc)-N323(sc)
loop (β17-β18)/ β20[a]	SB:D248(sc)-K268(sc)
loop (β17-β18)/ loop (β31-β32)[b]$_{D3}$	SB:D248(sc)-K418(sc), HB:G249(bb)-W417(bb)
Domain 3	
β25/β28[a]	SB:R346(sc)-E381(sc), R347(sc)-E384(sc), R347(sc)-E386(sc),VdW:V349(bb)-E386(bb), L350(bb)-R387(bb), W383(bb)-Arg346(bb)
loop (β28-β29)/loop (β32-β33)[a]	SB:D392(sc)-K424(sc), HB:K424(bb)-G389(bb), A425(bb)-Q388(bb)
β28/β33 [a]	SB:E384(sc)-R430(sc)
loop (β29-β30) /loop(β33-β34)[a]	SB:H400(sc)-E437(sc)
β29/β32 [b]	HB :L423(bb)-H393(bb)
β29/(α5)[b]	Π:H393(sc)-Cu463-H450(sc)
β34/β35[a]	SB:R440(sc)-E460(sc), HB:V461(bb)-G439(sc)
β27/β35[a]	SB: K374(sc)-E460(sc), HB: L373(bb)-E460bb), G375(bb)-E460(bb)

HB, hydrogen bond; SB, salt bridge; VdW, van der Waals interactions; Π, pi interactions; sc, side-chain; bb, backbone. α2-α3-helix$_{D1-D2}$ = linker between the domain 1 and 2, loop(β24-β25)$_{D2-D3}$= linker between the domain 2 and 3. Found for both systems[a], found only for holo*Tth*-MCO[b].
